# 6-gingerol and its derivatives inhibit *Helicobacter pylori*-induced gastric mucosal inflammation and improve gastrin and somatostatin secretion

**DOI:** 10.3389/fmicb.2024.1451563

**Published:** 2024-08-21

**Authors:** Jiali Qian, Zhennan Li, Jinhui Wang, Yuxian Lin, Yingcong Yu

**Affiliations:** ^1^The Third Affiliated Hospital of Shanghai University, Wenzhou People’s Hospital, Wenzhou, China; ^2^Department of Gastroenterology, Sir Run Run Shaw Hospital Affiliated to Zhejiang University School of Medicine, Hangzhou, Zhejiang, China; ^3^School of Medicine, Shanghai University, Shanghai, China; ^4^The First Affiliated Hospital of Wenzhou Medical University, Wenzhou, China; ^5^School of Pharmacy, Collaborative Innovation Center of Advanced Drug Delivery System and Biotech Drugs in Universities of Shandong, Key Laboratory of Molecular Pharmacology and Drug Evaluation (Yantai University), Ministry of Education, Yantai University, Yantai, China; ^6^The Third Clinical Institute Affiliated to Wenzhou Medical University, Wenzhou, China

**Keywords:** *Helicobacter pylori*, 6-gingerol, MIC, gastrin, somatostatin, IFN-γ, IL-4, IL-8

## Abstract

The resistance of *Helicobacter pylori* (*H. pylori*) has increased in recent years, prompting a trend in the research and development of new drugs. In our study, three derivatives (JF-1, JF-2, and JF-3) were synthesized using 6-gingerol as the main component, while JF-4, containing both 6-gingerol and 6-shogaol as the main components, was extracted from dried ginger. The minimum inhibitory concentrations (MICs), determined using the ratio dilution method, were 80 μg/mL for JF-1, 40 μg/mL for JF-2, 30 μg/mL for JF-3, 40 μg/mL for JF-4, 60 μg/mL for 6-gingerol standard (SS), and 0.03 μg/mL for amoxicillin (AMX). After treating *H. pylori*-infected mice, the inflammation of the gastric mucosa was suppressed. The eradication rate of *H. pylori* was 16.7% of JF-3 low-dose treatment (LDT), 25.0% of JF-3 high-dose treatment (HDT), 16.7% of JF-4 LDT, 16.7% of JF-4 HDT, 30% of SS LDT, 50% of SS HDT, and 36.4% of the positive control group (PCG). The levels of gastrin, somatostatin (SST), IFN-γ, IL-4, and IL-8 were significantly recovered in the JF-3 and JF-4 administration groups, but the effect was stronger in the high-dose group. These results demonstrate that 6-gingerol and its derivatives have significant anti-*Helicobacter pylori* effects and are promising potential treatments for *H. pylori* infection.

## 1 Introduction

*Helicobacter pylori* (*H. pylori*) is a common human pathogen that selectively colonizes human gastric epithelial cells (Sjomina et al., 2018). The average prevalence of *H. pylori* in China is 44.2%, which means that approximately 589 million people are infected (McColl, 2010). The *H. pylori* infection can cause varying degrees of intragastric disorders, including dyspepsia (5–10%), chronic gastritis (90%), peptic ulcers (15–20%), and gastric malignancies (1%) (Ding et al., 2021). This infection is also associated with extragastric disorders, such as inflammatory bowel disease, gastroesophageal reflux disease, non-alcoholic fatty liver disease, hepatocellular carcinoma, cholelithiasis, and cholecystitis (Santos et al., 2020).

The International Agency for Research on Cancer (IARC) classified *H. pylori* as a class I carcinogen in 1994, and an early intervention to eradicate *H. pylori* can reverse chronic active gastritis. Currently, the first-line treatment regimen for the eradication of *H. pylori* infection is the quadruple therapy consisting of bismuth+proton-pump inhibitors (PPI) + 2 antimicrobial drugs. There are other treatments, which include standard triple therapies, high-dose dual therapies, sequential therapies, traditional Chinese medicine (TCM) treatments, the use of probiotics, and *H. pylori* vaccine development (Sugano et al., 2015; Sulo and Šipková, 2021; Yang and Hu, 2021). However, *H. pylori* resistance to antibiotics has been increasing globally due to their widespread use, with primary and secondary resistance rates of clarithromycin, metronidazole, and levofloxacin being more than 15% in all regions monitored by the World Health Organization (WHO) (Yoshii et al., 2020). In addition, there are common side effects of standard quadruple therapy, which include abdominal pain, nausea and vomiting, rash, abdominal distension, and dizziness (Yan et al., 2024). These side effects also cause significant discomfort for patients during treatment. Therefore, developing new drugs is an effective way to alleviate the challenge of microbial resistance.

Ginger is a perennial herbaceous plant of the genus *Zingiber* in the family Zingiberaceae. It is a traditional Chinese medicinal and food plant. Gingerol is the main active ingredient in dried ginger, which also contains shogaol, gingerone, and other spicy substances (Liu et al., 2019). In 1969, Connell and Sutherland isolated gingerol and confirmed that the content of 6-gingerol (6-G) accounted for approximately 75% of the gingerol. Gingerol has a variety of biological properties, such as antitumor, anti-inflammatory, antioxidant, anticoagulant, analgesic, antiemetic, hypoglycemic, hypolipidemic, cardiovascular, and neuroprotective effects (Wang et al., 2015), and has been widely used in the alleviation and treatment of many diseases (Chey et al., 2017; Liu et al., 2018). Several studies have shown that 6-gingerol also has antimicrobial (Fallone et al., 2016), antiviral (Haikh and Fallone, 2016; Ko S. W. et al., 2019), and antiparasitic activities (Graham et al., 2018; Li et al., 2019), which may be related to the inhibition of bacterial ATP synthase binding, thereby reducing bacterial biofilm formation and virulence (Sun, 2011; Smith et al., 2017).

**Figure 1:**
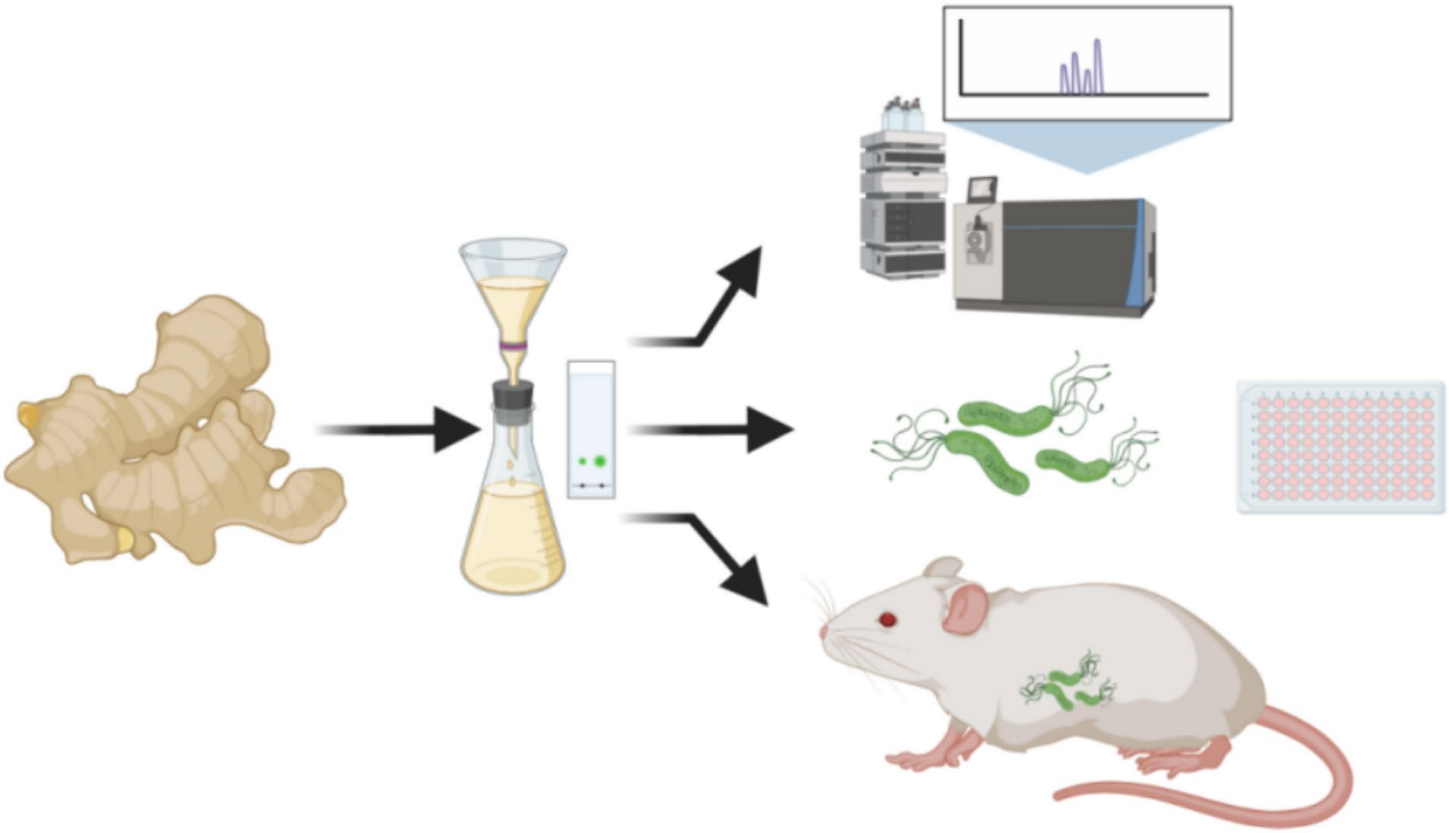
Dried ginger oil was extracted and purified for the LC–MS analysis, *H. pylori* antibacterial assay, and *in vivo* animal treatment experiment. (Images produced by https://www.biorender.com/).

There are no studies on gingerols and their derivatives against *H. pylori*. To explore the resistance of 6-gingerol to *H. pylori* and to develop new natural product drugs, in the early stage of this study, self-made dried ginger slices were optimized according to the orthogonal test method, and 6-gingerol purified (JF-4) was obtained after isolation and purification. The main components of JF-4 are 6-gingerol and 6-shogaol, as described in the LC–MS database. However, 6-gingerol is chemically unstable and prone to degradation and dehydration (Hu et al., 2017). To solve the instability of 6-gingerol and maintain good properties, three 6-gingerol derivatives, JF-1, JF-2, and JF-3, were synthesized based on the retention of the active group of 6-gingerol. This synthesis improved the stability, solubility, and potency of 6-gingerol and helped it play its active role better. The results showed that 6-gingerol oil purification and derivatives had anti-*H. pylori* infection *in vitro* and *in vivo* (Figure 1).

## 2 Materials and methods

### 2.1 Chemicals

Analytical grade chemicals including 4% paraformaldehyde, dimethyl sulfoxide (DMSO), 6-gingerol standard (SS), clarithromycin, amoxicillin, omeprazole, bismuth potassium citrate, and anhydrous ethanol were obtained from Beijing Solarbio Science & Technology Co., Ltd., Shanghai Macklin Biochemical Co. Ltd., and Shanghai Aladdin Bio-Chem Technology Co., LTD. Tianjin Beichen Fangzheng Reagent Factory was the source of solvents and reagents.

### 2.2 Purification and synthesis of 6-gingerol derivatives

Fresh ginger slices were steamed for 30 min and were baked in an electric blast drying oven for 60 min at a temperature of 50°C. Then, the slices were dried and ground into a powder, followed by ultrasonic extraction using 10 times the volume of 80% ethanol for 30 min at a temperature of 60°C. The extraction was filtered using a 0.22-μm microporous filter membrane and then was freeze-dried to obtain the gingerol extract. Furthermore, 400 g of 100–200 mesh silica gel was used for chromatography, being eluted with a gradient mixture of petroleum ether and ethyl acetate at a ratio of 5:1. Afterward, the mixture underwent rotary evaporation and concentration to obtain the purified 6-gingerol.

JF-1, JF-2, and JF-3 were designed and synthesized by Dr. Huang Xianfeng’s team at Changzhou University, with the structural formula as shown in Table 1.

**Table 1:** The structural formula of 6-gingerol derivatives.

Serial number	JF-1	JF-2	JF-3
Structural formula (chemistry)	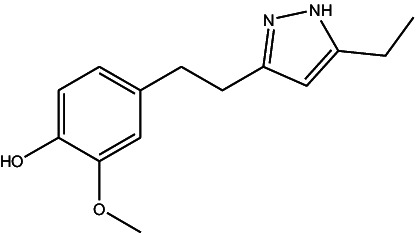	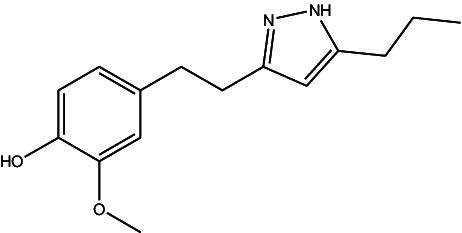	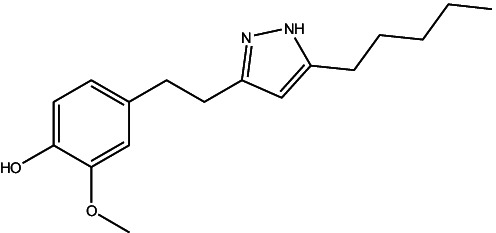
Molecular mass (g/mol)	246	260	288

The presence of N atoms in the molecule increases water solubility and facilitates administration as a salt.

### 2.3 Minimum anti-*Helicobacter pylori* concentration of dried ginger extracts

*Helicobacter pylori* GDMCC 1.1820 was purchased from Guangzhou Di Jing Microbiology Co. The ratio dilution method was as follows: Three novel compounds (JF-1, JF-2, and JF-3), 6-gingerol purifier (JF-4), control drugs (amoxicillin and AMX), and SS were prepared as a masterbatch in dimethylsulfoxide (DMSO) at a concentration of 3,200 μg/mL, and the masterbatch was filtered and sterilized using a 0.22-μm filter. Then, 160 μL of an *H. pylori* culture solution was added to the wells of column 1 of a 96-well plate, followed by the addition of 40 μL of the masterbatch of drugs. Furthermore, 100 μL of the *H. pylori* culture solution was added to the wells of columns 2 through 12. Then, 100 μL of the mixture from column 1 was pipetted into column 2, 100 μL of the mixture from column 2 was pipetted into column 3, and finally, the mixture was doubled and diluted to column 12 in this order. The mixture from column 12, amounting to 100 μL, was discarded, and 100 μL of the abovementioned *H. pylori* bacterial solution was added to columns 1 through 12. The final concentrations of the drug in the wells of columns 1–12 were 320 μg/mL, 160 μg/mL, 80 μg/mL, 40 μg/mL, 20 μg/mL, 10 μg/mL, 5 μg/mL, 2.5 μg/mL, 1.25 μg/mL, 0.625 μg/mL, 0.3125 μg/mL, and 0.15625 μg/mL. In addition, the negative control (with or without the bacteria solution) and the positive control (with or without the bacteria solution) were set up. The 96-well plate was incubated at 37°C in a microaerobic environment within a constant temperature incubator for 48 to 96 h. No precipitation at the bottom and a clear solution without reddening indicated that bacteria did not grow. The lowest drug concentration was the minimum inhibitory concentration (MIC).

### 2.4 Animal grouping and modeling

Male SPF BALB/c mice, 6–8 weeks old and weighing 18–22 g, were purchased from Changzhou Cavins Laboratory Animal Co. Ltd. The mice were acclimatized and fed for 1 week before the formal experiments, and during this period, the mice were free to eat and move around. The animal experiments were approved by the Ethics Committee of the Animal Experimentation Center of Yantai University as required.

In this study, the mice were randomly divided into nine groups of 10–12 mice each: normal control group (NCG), *H. pylori* model group (HMG), *H. pylori* model group + JF-3 low-dose treatment (LDT) group (50 mg/kg, JF-3 LDT), *H. pylori* model group + JF-3 high-dose treatment (HDT) group (200 mg/kg, JF-3 HDT), *H. pylori* model group + JF-4 low-dose treatment group (50 mg/kg, JF-4 LDT), *H. pylori* model group + JF-4 high-dose treatment group (200 mg/kg, JF-4 HDT), *H. pylori* model group +6-G standard low-dose treatment group (50 mg/kg, SS LDT), *H. pylori* model group +6-G standard high-dose treatment group (200 mg/kg, SS HDT), and positive control group (PCG, standard quadruple-drug treatment).

The mice model of the *H. pylori*-infected gastritis group was constructed as follows: After 1 week of acclimatization, the mice were randomly divided into normal control and *H. pylori*-infected groups. The *H. pylori*-infected group was given 0.3 mL of *H. pylori* suspension (9 × 10^8^ CFU/mL) by gavage every other day, and 0.3 mL of saline was given to the control group by gavage. Before the gavage, a period of 12 h of fasting, including water fasting, was imposed, and 4 h of free feeding was performed after the gavage. A total of five inoculations were performed (0.3 mL of 5% NaHCO_3_ was given to the mice 0.5 h before the *H. pylori* gavage to increase the rate of *H. pylori* infection). After 4 weeks of continued feeding, eight mice in the infected group were randomly executed and subjected to a rapid urease test and staining of pathological sections (Warthin-Starry silver staining + HE staining) to detect whether they were infected with *H. pylori* or not. A positive rate of ≥75% was considered successful.

After confirming the success of the model, the mice infected with *H. pylori* were randomly divided into eight groups and treated with corresponding drugs for 2 weeks: the *H. pylori* model group: 0.3 mL/ sterile saline was given twice a day; the standard quadruple-drug treatment group: amoxicillin 150 mg/kg + clarithromycin 75 mg/kg + omeprazole 6 mg/kg + bismuth potassium citrate 33 mg/kg was given twice a day; the high-dose and low-dose groups: the mice were treated with three drugs, JF-3, JF-4, and 6-G standard, with corresponding doses, a low dose of 50 mg/kg and a high dose of 200 mg/kg, by gavage twice a day; and the normal control group: 0.3 mL/ sterile saline was given by gavage twice a day. All mice were killed 4 weeks after the withdrawal of drugs.

### 2.5 Evaluation of urease activity

The appropriate amount of urease reaction solution was taken in Eppendorf (EP) tubes. Then, a small piece of mucosa from the gastric sinus and gastric body of the mice was clipped and placed in the reaction solution. The tubes were sealed and left to sit for 10 min to observe for any color change. If the color change was minimal, the tubes were placed in a 37°C incubator and the color change was observed after 12 h. A 0.2-mL suspension of *H. pylori* was taken for comparison in the same way.

### 2.6 ELISA (double antibody sandwich assay) for inflammation-related factors

For the serum, blood was collected from the eyeballs of the mice immediately after execution, allowed to sit at room temperature for 2 h, and then centrifuged at 1000 rpm for 15 min at 2–8°C. A portion of the supernatant was used for the test. For the gastric mucosa, a portion of the gastric mucosa tissue of the mice was collected, washed with PBS to remove blood stains, cut into small pieces, and put into a tissue grinder. Then, 1 mL of PBS was added to create a homogenate, which was then centrifuged at 5000 rpm for 5 min at 2–8°C. The supernatant was used for the experiment. Standard wells and sample wells were set up separately. Then, 100 μL of the standard or sample to be tested was added to each well, fully mixed and covered with a plate sticker, and was incubated at 37°C for 2 h. The liquid was discarded, and the wells were shaken dry; 100 μL of biotin-labeled antibody was added to each well, covered with a new plate sticker, and incubated at 37°C for 1 h. Then, the liquid in the wells was discarded, the wells were shaken dry, and the plate was washed three times for 2–3 min each. Furthermore, 100 μL of horseradish peroxidase-labeled working solution was added to each well, covered with a new plate seal, and incubated at 37°C for 1 h. The liquid in the wells was discarded, the wells were shaken dry, and the plate was washed five times for 2–3 min. A volume of 100 μL of the substrate solution (TMB) was added to each well, and the reaction was terminated by adding 50 μL of the termination solution to each reaction well after it was made to stand for 20 min at 37°C in a dark environment. The optical density (OD) of each well was measured with an enzyme counter at 450 nm within 5 min after the reaction termination. The concentration fitting curve was calculated according to the OD value of the standard, and the actual concentration was calculated according to the fitting curve.

### 2.7 Statistical methods

The data were statistically analyzed using SPSS 26.0 statistical software and presented as mean ± standard. The data between two groups were analyzed by performing an independent samples *t*-test, comparisons between multiple groups were analyzed using a one-way ANOVA, two-by-two comparisons between groups were analyzed by performing Dunnett’s *t*-test, and variance disagreement was analyzed by performing Tamhane’s T2 test. A *p*-value of <0.05 indicated that the difference was statistically significant.

## 3 Results

### 3.1 Effect of 6-gingerol purification

**Figure 2:**
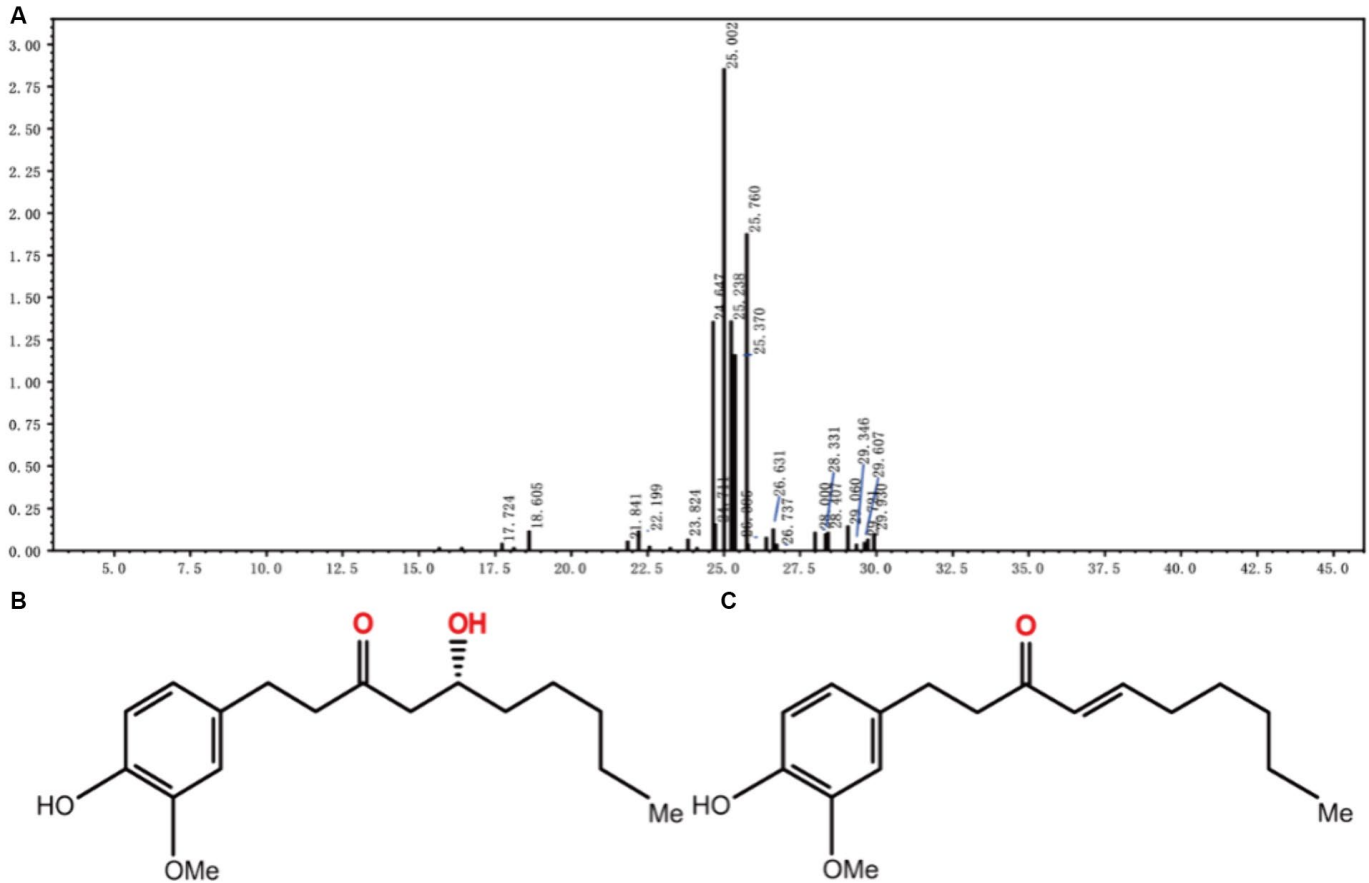
LC–MS mass spectra of JF-4 **(A)**. Schematic representation of the molecular structure of 6-gingerol **(B)**. Schematic representation of the molecular structure of 6-shogaol **(C)**.

A 5:1 gradient of petroleum ether to ethyl acetate was used as the mobile phase for elution. The light brown oily liquid obtained by column chromatography (Figure 2A) was 6-gingerol oil. Kim J. et al. (2022) found the main components of dried ginger extract, including substances suchas 6-gingerol (Figure 2B), 6-shogal(Figure 2C), 8-gingerol, and 10-gingerol, and verified that dried ginger extract can enhance immunity after influenza vaccination. Other studies have shown (Mustafa et al., 2019; Kim J. Y. et al., 2022; Mustafa and Chin, 2023) that different extraction methods, freshness, species, and parts of the plant have an effect on the extraction results, but most of the constituents are gingerols, shogaols, and some alkenes. Ginger extracts have been reported in the research fields for their anti-inflammatory (Ballester et al., 2022), antioxidant (Mustafa and Chin, 2023), antimicrobial (He et al., 2023), and anticancer properties (Mahomoodally et al., 2019), and 6-gingerol can be converted to 6-gingerol by subcritical water extraction (Ko M. J. et al., 2019). Some studies have shown that 6-gingerol inhibits adipocyte hypertrophy and proliferation, improves metabolic disorders (Cheng et al., 2022), and ameliorates fatty liver degeneration, inflammation, and oxidative stress in mice by activating the LKB1/AMPK signaling pathway (Liu et al., 2023). It also ameliorates multiple disorders, such as brain injury (Tang et al., 2022), lung injury (Li et al., 2024), myocardial ischemia (Lv et al., 2021), and other multiple diseases.

### 3.2 *In vitro* minimum inhibitory concentration (MIC)

The results of the doubled dilution method were as follows: three novel compounds, JF-1, JF-2, and JF-3, and the purified product (JF-4) were found to be bacteriostatic against *H. pylori in vitro*, with average minimum inhibitory concentrations of 80 μg/mL, 40 μg/mL, 20 μg/mL, and 40 μg/mL, respectively. The minimum inhibitory concentration of the SS was 40 μg/mL, and the minimum inhibitory concentration of amoxicillin was 0.03 μg/mL. The antibacterial effects of JF-1 and JF-2 were weak, and no animal experiments were performed (Table 2).

**Table 2:** MIC values (μg/mL) of *in vitro* anti-*H. pylori* for each sample of the multiplicative dilution method.

MIC values (μg/mL) for each sample in the three experiments							
	JF-1	JF-2	JF-3	JF-4	SS	AMX	DMSO
1	80	40	20	40	40	Negative	160
2	40	40	20	20	40	0.02	160
3	80	80	20	40	40	0.04	160
Result	80	40	20	40	40	0.03	160

As *H. pylori* did not grow in the amoxicillin culture solution at a concentration of 0.15625 μg/mL in the first experiment, the concentration of amoxicillin was further reduced in the second and third experiments.

The present study demonstrated that dried ginger extract has good anti-*H. pylori* properties and has potential therapeutic effects against *H. pylori* infection. In gingerol antimicrobial-related studies, dried ginger extract inhibited the biofilm formation of *Candida albicans*, reduced its virulence, and prevented mycelial growth and aggregation (Lee et al., 2018). Hughes et al. (2021) reported that it had an ATP synthase inhibitory effect on *E. coli*. According to Wang et al. (2020) 6-gingerol was found to affect the composition of the intestinal microbiota and was able to significantly increase the abundance of probiotics, such as bifidobacteria and enterococci. Finally, 6-gingerol was found to be a potential prebiotic (Wang et al., 2020).

### 3.3 Modeling

**Figure 3:**
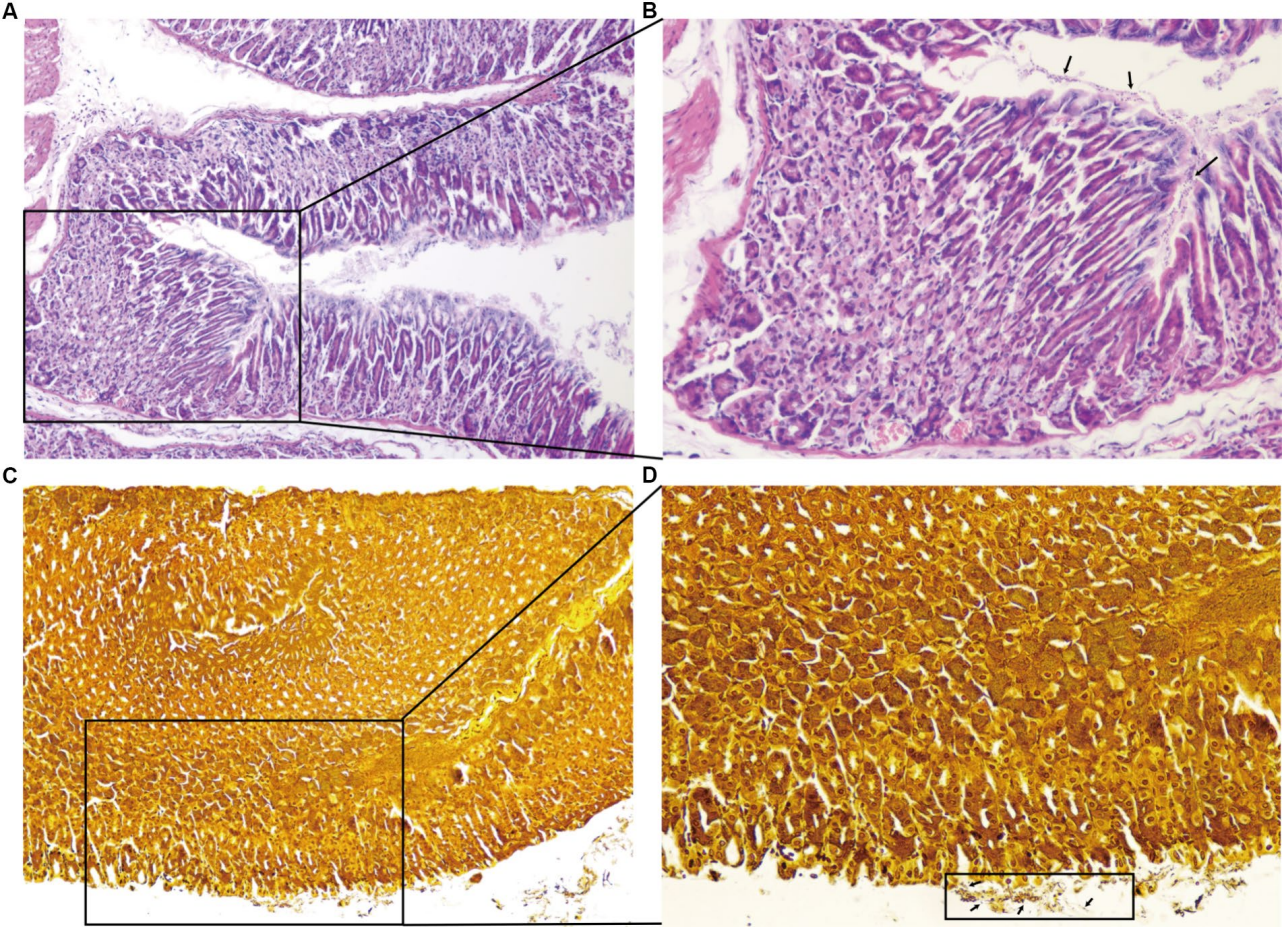
Results of the pathological sections of the gastric antrum tissue. HE staining showing neutrophilic infiltration, lymphocytes, and other inflammatory cell infiltration (**A** ×100,**B** ×200). Warthin–Starry silver staining ×200, a large number of *H. pylori* colonization on the surface of gastric mucosal epithelial cells and the bottom layer of gastric mucus (**C** ×100,**D** ×200).

There were 10 mice in the normal control group with good growth activities, and there were 98 mice in the *H. pylori*-infected group, some of which showed loss of appetite, depression, and unresponsiveness during the feeding process. Microscopic examination of the gastric mucosa in mice revealed infiltration of neutrophils, lymphocytes, and other inflammatory cells. *H. pylori* colonization was also evident in the pathological sections (Figure 3).

The *H. pylori* infection causes intense lymphocyte infiltration in the stomach, and CD8 + T cells infiltrate the gastric mucosa soon after the infection, playing a crucial role in controlling the pathogen by performing antigen-specific effector properties (Koch et al., 2023). Moreover, it has been demonstrated that *H. pylori* cleaves the epithelial cell attachment adhesion molecule to disrupt gastric epithelial integrity (Marques et al., 2021).

### 3.4 *Helicobacter pylori* decontamination rate results

As shown in Table 3, it was observed that all mice in the normal control group tested negative for gastric mucosal urease and showed no abnormalities during the pathological examination. On the other hand, 90.9% of mice in the *H. pylori* model group tested positive for gastric mucosal urease, all of which exhibited abnormalities during the pathological examination and were infected with *H. pylori*. The eradication rate was calculated as the number of mice with both tests negative in each group divided by the total number of mice in each group, and the eradication rates for each drug were 25.0% (JF-3 HDT), 16.7% (JF-3 LDT), 16.7% (JF-4 HDT), 16.7% (JF-4 LDT), 50% (SS HDT), 30% (SS LDT), and 36.4% (PCG).

**Table 3:** Results of the *H. pylori* sterilization rate.

Groups	Number of mice	Number of urease-negative	Number of pathology *H. pylori* (−)	Number of mice negative for both tests	Eradication rate
NCG	10	10 (100%)	10 (100%)	10 (100%)	–
MG	11	1 (9.1%)	0 (0%)	0 (0%)	0%
JF-3 HDT	12	8 (66.7%)	4 (33.3%)	3 (25.0%)	25.0%
JF-3 LDT	12	5 (41.7%)	2 (16.7%)	2 (16.7%)	16.7%
JF-4 HDT	12	8 (66.7%)	4 (33.3%)	2 (16.6%)	16.7%
JF-4 LDT	12	5 (41.7%)	6 (50.0%)	2 (16.7%)	16.7%
SS HDT	10	9 (90.0%)	6 (60.0%)	5 (50%)	50%
SS LDT	10	6 (60.0%)	5 (50.0%)	3 (30%)	30%
PCG	11	7 (63.6%)	5 (45.6%)	4 (36.4%)	36.4%

### 3.5 Pathological findings

**Figure 4:**
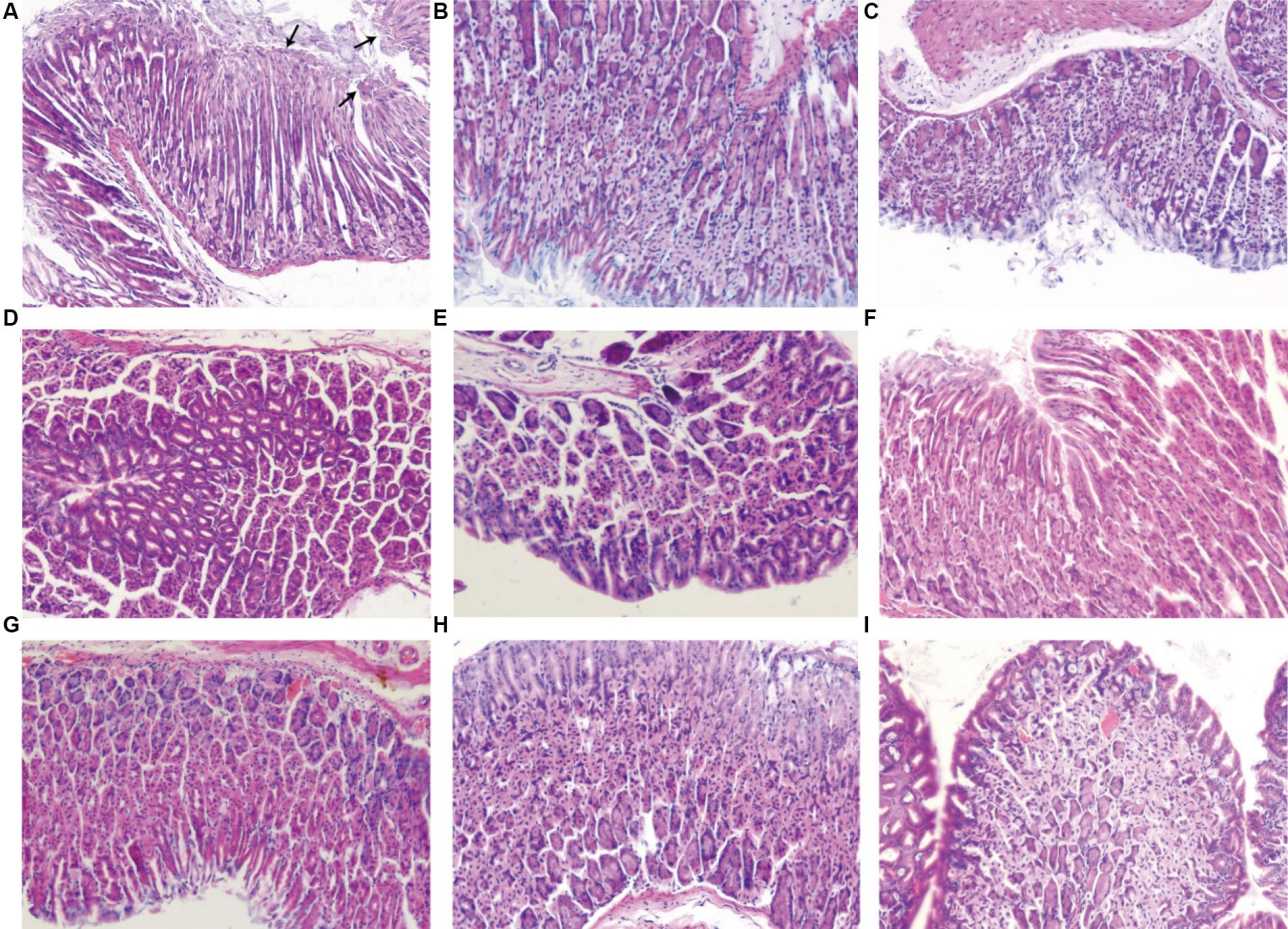
Results of the pathological sections of the gastric antrum tissue. In the *H. pylori* model group **(A)**, neutrophil infiltration was observed in the background of chronic inflammation, and short rod-shaped *H. pylori* fixation was observed at the arrow under the microscope. Normal control group **(B)**. Positive control group **(C)**. JF-3 high-dose group **(D)** and low-dose group **(G)**. JF-4 high-dose group **(E)** and low-dose group **(H)**. SS high-dose group **(F)** and low-dose group **(I)**.

As shown in Figure 4 and Table 4, compared to the normal control group, chronic inflammation of the gastric mucosa in mice from the *H. pylori* model group was more severe; the activity was obvious and the difference was statistically significant (*p* < 0.01; *p* < 0.001). The *H. pylori* infection was observed in all of the *H. pylori* model groups. Compared with the HMG, chronic inflammation of the gastric mucosa in mice was partially improved after drug treatment, but none of the differences were statistically significant. Compared with the HMG, the inflammatory activity and *H. pylori* infection of the gastric mucosa in mice were significantly improved (neutrophil infiltration was reduced) after 14 days of treatment with JF-3, JF-4, and SS, as well as after treatment with the PCG (*p* < 0.01). It was shown (Tian et al., 2022) that 6-gingerol attenuates Dextran Sulfate Sodium Salt (DSS)-induced colitis, mainly by interfering with antioxidant and anti-inflammatory pathways and the ratio of the thick-walled bacillus phylum/anabolic bacillus phylum. It also has the ability to inhibit the activity of *Staphylococcus aureus* and *Escherichia coli*, which suggests a good antimicrobial effect (Ahad et al., 2023). 6-gingerol has also been shown to regulate the structure and metabolites of digestive flora (Shen et al., 2022). Li et al. (2017) demonstrated that 6-gingerol could protect against intestinal barrier damage by inhibiting p38 MAPK to NF-κB signaling.

**Table 4:** Pathologic results of HE staining.

Groups	Number of mice	Chronic inflammation ( $\bar{x}$ ± s)	Activity ( $\bar{x}$ ± s)	*H. pylori* infection status			
				0	+	++	*H. pylori* ( $\bar{x}$ ± s)
NCG	10	1.50 ± 0.53	0.20 ± 0.42	10	0	0	0
MG	11	2.00 ± 0.00**	1.36 ± 0.50***	0	4	7	1.64 ± 0.50***
JF-3 HDT	12	2.00 ± 0.43	0.58 ± 0.67##	2	6	4	1.17 ± 0.72
JF-3 LDT	12	1.83 ± 0.39	0.50 ± 0.52##	4	6	2	0.83 ± 0.72#
JF-4 HDT	12	1.92 ± 0.29	0.33 ± 0.49###	4	8	0	0.67 ± 0.49###
JF-4 LDT	12	1.75 ± 0.45	0.42 ± 0.51###	6	5	1	0.58 ± 0.67###
SS HDT	10	1.90 ± 0.32	0.10 ± 0.32###	5	5	0	0.50 ± 0.53###
SS LDT	10	1.70 ± 0.48	0.30 ± 0.48###	5	5	0	0.50 ± 0.53###
PCG	11	1.91 ± 0.30	0.55 ± 0.52##	4	4	3	0.91 ± 0.83#

**p < 0.01 versus the normal control group; ***p < 0.001 versus the normal control group; #*p* < 0.05 versus the *H. pylori* model group; ##*p* < 0.01 versus the *H. pylori* model group; ###*p* < 0.001 versus the *H. pylori* model group.

### 3.6 Expression results of gastrin and somatostatin in serum

**Figure 5:**
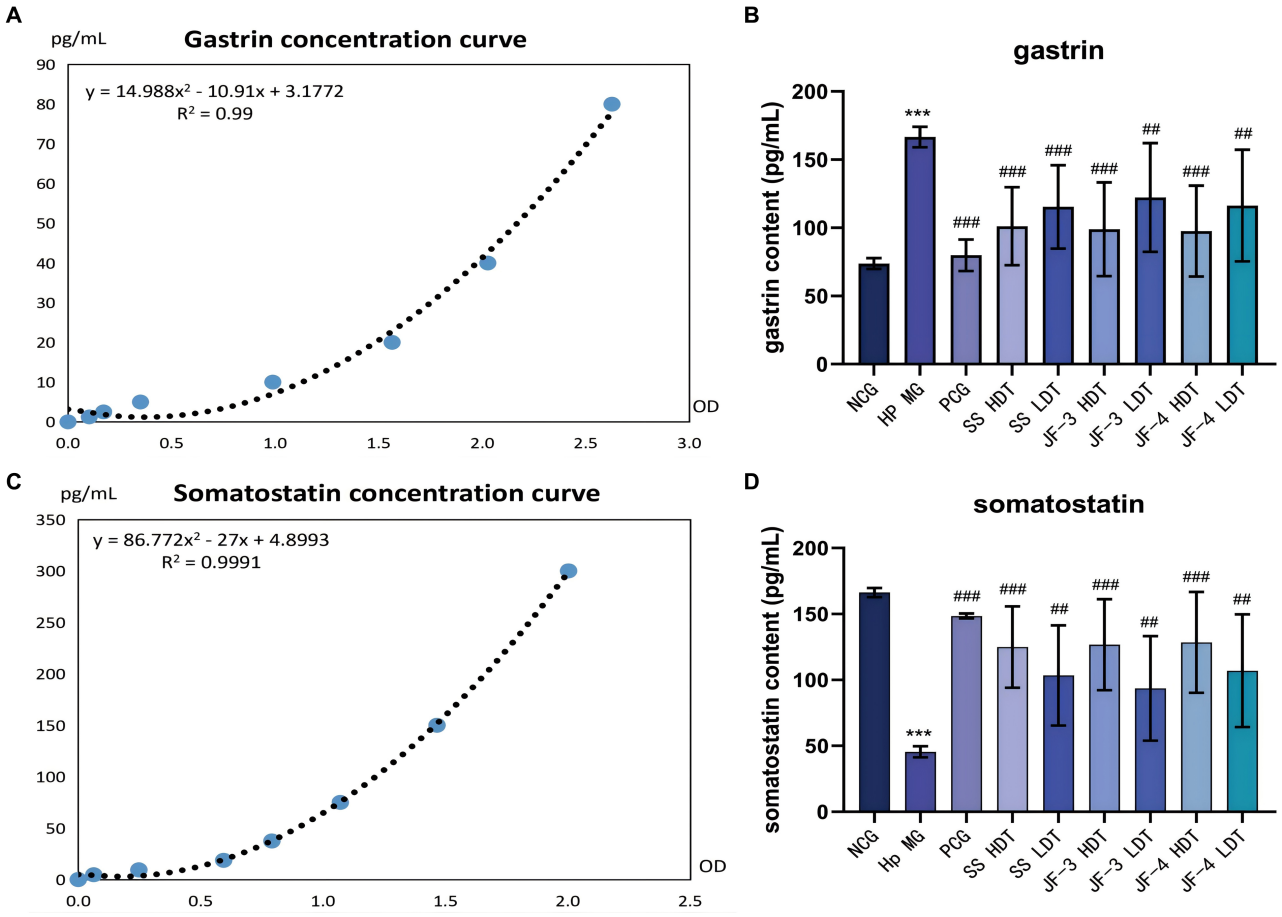
Expression results of gastrin and somatostatin in the serum. Fitting curve of gastrin concentration (dilution of 10 times) **(A)**. Concentration of gastrin in the serum of different groups of mice **(B)**. Fitting curve of somatostatin concentration **(C)**. Concentration of somatostatin in the serum of different groups of mice **(D)**. *P < 0.05 vs normal control group; **P < 0.01 vs normal control group; ***P < 0.001 vs normal control group; #*P* < 0.05 vs Hp model group; ##*P* < 0.01 vs Hp model group; ###*P* < 0.001 vs Hp model group.

Serum gastrin and somatostatin (SST) concentrations were significantly changed in the *H. pylori*-infected mice compared with the normal control. After 14 days of treatment with JF-3, JF-4, and SS, it was observed that the treatment with gastrin and somatostatin resulted in varying degrees of recovery. When comparing different doses of the same therapeutic drug, the changes caused by the high dose were found to be more significant; in particular, the high dose of JF-3 had a statistically significant effect relative to the low dose of JF-4 (Figure 5 and Table 5).

**Table 5:** Serum levels of gastrin and somatostatins in the mice.

Groups	Number of mice	Gastrin		Somatostatin	
		OD value	Concentration (pg/mL)	OD value	Concentration (pg/mL)
NCG	10	1.01 ± 0.02	73.69 ± 4.00	1.53 ± 0.01	166.27 ± 3.46
MG	11	1.38 ± 0.02	166.53 ± 7.51***	0.86 ± 0.03	45.47 ± 4.23***
JF-3 HDT	12	1.11 ± 0.14	98.98 ± 34.33###	1.34 ± 0.18	126.68 ± 34.51###
JF-3 LDT	12	1.21 ± 0.16	122.29 ± 39.83##	1.16 ± 0.22	93.56 ± 39.67##
JF-4 HDT	12	1.11 ± 0.14	97.56 ± 33.37###	1.34 ± 0.20	128.50 ± 38.23###
JF-4 LDT	12	1.18 ± 0.17	116.32 ± 40.93##	1.23 ± 0.23	106.99 ± 42.76##
SS HDT	10	1.13 ± 0.12	101.13 ± 28.62###	1.33 ± 0.16	124.91 ± 30.84###
SS LDT	10	1.19 ± 0.13	115.37 ± 30.56###	1.21 ± 0.21	103.42 ± 37.97##
PCG	11	1.04 ± 0.06	79.87 ± 11.62###	1.45 ± 0.01	148.48 ± 1.95###

***p < 0.001 versus the normal control group; ##*p* < 0.01 versus the *H. pylori* model group; ###*p* < 0.001 versus the *H. pylori* model group.

Gastrin primarily regulates gastric acid secretion and cell proliferation in the acid-secreting mucosa. Chronic gastritis caused by the *H. pylori* infection usually shows high levels of gastrin expression (Rehfeld, 2021; Veysey-Smith et al., 2021). Somatostatin (SST) is a major inhibitor of gastric acid secretion, which can inhibit the gastrin secretion of G cells and the gastric acid secretion of parietal cells (Schubert and Rehfeld, 2019). SST also has anti-proliferative and pro-apoptotic properties and has been listed as a first-line anticancer drug in clinical practice (Shamsi et al., 2021). The imbalance between somatostatin and gastrin further leads to chronic inflammation and the occurrence of gastric cancer. The metabolites of *H. pylori* increase the pH value of the gastric epithelial surface, and hence, the feedback effect of gastric acid on gastrin is disturbed. Furthermore, TNF α, IFN - gamma, and cytokine stimulation, such as IL-8 G cells, secrete gastric elements (Maciorkowska et al., 2006). In addition, there was a decrease in the number of D cells and in the expression level of somatostatin (Kim et al., 2020; Kunovsky et al., 2021).

The study’s findings indicated that the serum gastrin content increased and the somatostatin content decreased in the mice infected with *H. pylori*. However, after treatment with 6-gingerol and its derivatives, the serum gastrin and somatostatin concentrations significantly recovered. The *H. pylori* infection causes an imbalance between somatostatin and gastrin, which promotes chronic inflammation and the development of gastric cancer. Gingerol treatment significantly enhances the secretion of gastrin and somatostatin, reducing the risk of peptic ulcer. This confirmed that gingerol, especially in the high-dose group, could be a potential agent for repairing gastric injury caused by *H. pylori*.

### 3.7 Results of IFN-γ, IL-4, and IL-8 expression levels in gastric mucosa tissues

**Figure 6:**
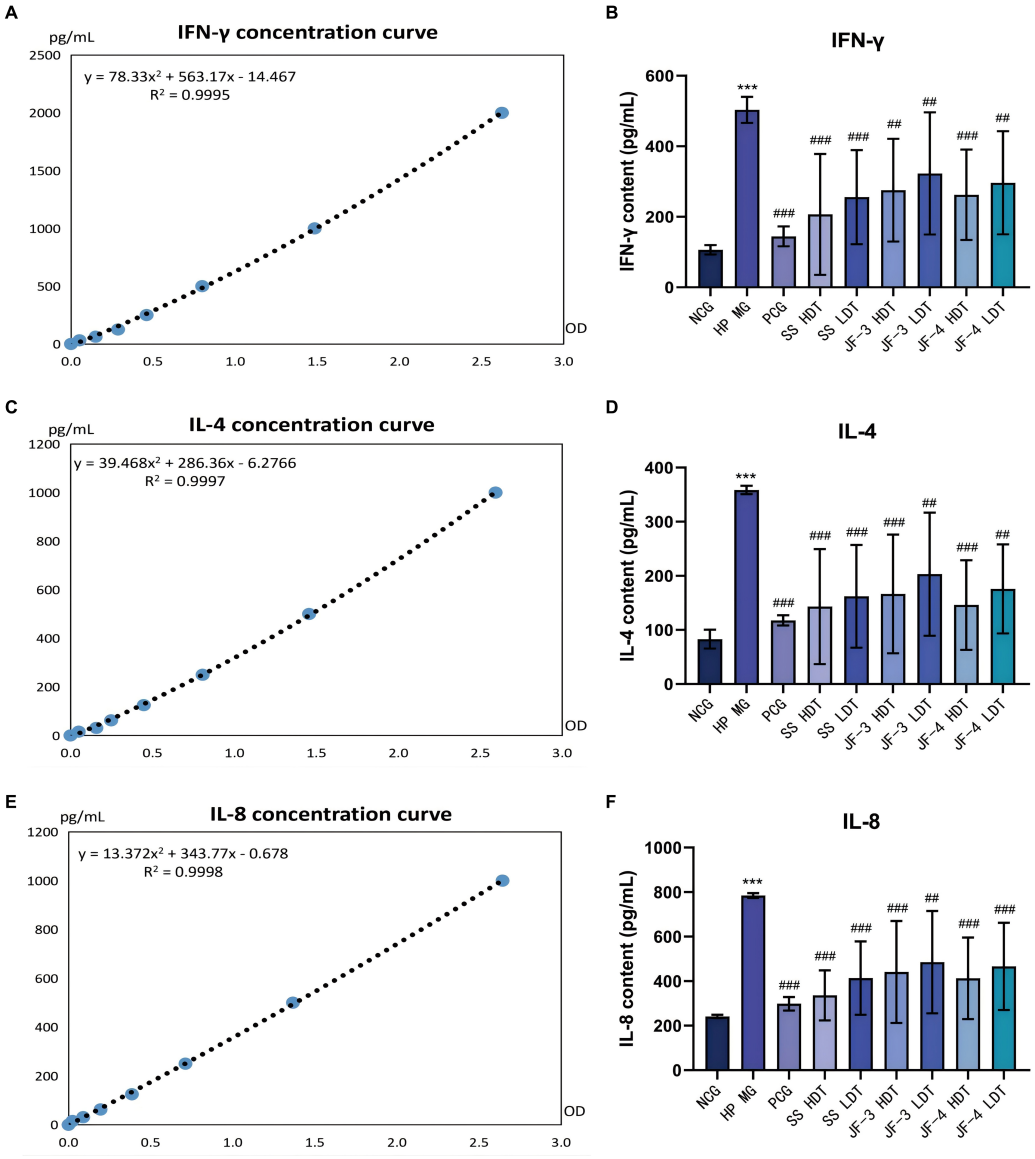
Expression results of IFN-γ, IL-4, and IL-8 in gastric mucosa tissues. IFN-γ concentration curves **(A)**, expression levels of IFN-γ in the gastric mucosa of different groups of mice **(B)**. IL-4 concentration curves **(C)**, expression levels of IL-4 in the gastric mucosa of different groups of mice **(D)**. IL-8 concentration curves **(E)**, expression levels of IL-8 in the gastric mucosa of different groups of mice **(F)**.

In the mice model of *H. pylori* infection, significantly changes were observed in the expression levels of IFN-γ, IL-4, and IL-8 in the gastric mucosa of the mice compared to those in the normal control group. Following the infection, there was a notable increase in the expression of IFN-γ, IL-4, and IL-8. After 14 days of treatment with JF-3, JF-4, and SS, the expression levels of IFN-γ, IL-4, and IL-8 were all reversed to varying degrees. During the comparison of different drug doses, it was observed that the expression levels of IFN-γ, IL-4, and IL-8 decreased more significantly after high-dose treatment as compared to low-dose treatment, although the difference was not statistically significant. After PCG treatment, the expression levels of IFN-γ, IL-4, and IL-8 decreased significantly (Figure 6 and Table 6).

**Table 6:** Levels of IFN-γ, IL-4, and IL-8 in the gastric mucosa of the mice.

Groups	Number of mice	IFN-γ		IL-4		IL-8	
		OD value	Concentration (pg/mL)	OD value	Concentration (pg/mL)	OD value	Concentration (pg/mL)
NCG	10	0.21 ± 0.02	106.40 ± 13.52	0.30 ± 0.06	82.96 ± 17.60	0.69 ± 0.02	241.46 ± 7.62
MG	11	0.82 ± 0.05	503.29 ± 37.09***	1.11 ± 0.02	358.63 ± 7.85***	2.11 ± 0.03	784.66 ± 10.75***
JF-3 HDT	12	0.48 ± 0.22	275.47 ± 145.75##	0.55 ± 0.32	166.48 ± 109.79###	1.21 ± 0.60	441.09 ± 228.96###
JF-3 LDT	12	0.55 ± 0.27	322.89 ± 173.21##	0.66 ± 0.34	203.01 ± 113.92##	1.33 ± 0.60	485.71 ± 230.03##
JF-4 HDT	12	0.46 ± 0.20	262.37 ± 128.26###	0.49 ± 0.25	146.02 ± 82.87###	1.14 ± 0.48	412.96 ± 183.04###
JF-4 LDT	12	0.51 ± 0.23	296.47 ± 146.42##	0.58 ± 0.25	175.85 ± 82.41##	1.28 ± 0.52	466.22 ± 195.52###
SS HDT	10	0.37 ± 0.27	206.69 ± 171.58###	0.48 ± 0.32	143.14 ± 106.33###	0.94 ± 0.30	335.94 ± 112.54###
SS LDT	10	0.45 ± 0.21	255.69 ± 133.58###	0.54 ± 0.29	162.31 ± 95.00###	1.15 ± 0.44	413.62 ± 165.05###
PCG	11	0.27 ± 0.05	144.42 ± 28.24###	0.41 ± 0.03	117.61 ± 9.50###	0.84 ± 0.08	298.56 ± 30.35###

***p < 0.001 versus the normal control group; ##*p* < 0.01 versus the *H. pylori* model group; ###*p* < 0.001 versus the *H. pylori* model group.

The results of several studies (Bagheri et al., 2018; Yang et al., 2020) have shown that, after *H. pylori* infection, the host’s Th1 immune response is enhanced and dominant, and the expression levels of inflammatory factors, such as INF-γ, IL-2, and TNF-α, are increased. The phosphorylation of AKT and STAT3 promotes differentiation and increases IFN-γ-producing T cells. ADM also induces macrophages to produce IL-12 and aggravates T-cell response (Kong et al., 2020), which further promotes the occurrence of *H. pylori*-associated gastritis. Blocking or knocking out IFN-γ can reduce the inflammatory response of gastric mucosa in mice infected with *H. pylori*. Multiple studies, carried out both domestically and internationally, have shown that CagA, which is produced by highly virulent strains of *H. pylori*, can trigger the NF-κB and AP-1 pathways in gastric epithelial cells and the nuclear heptose biosynthesis pathway in Lipopolysaccharide (LPS). This activation results in the expression of IL-8 (Stein et al., 2017; Zhang et al., 2019; Yu et al., 2020).

After treatment with 6-gingerol and JF-3, the expression levels of IFN-γ, IL-4, and IL-8 decreased significantly. This suggests that 6-gingerol and its derivatives may help restore the expression levels of INF-γ and IL-4, ultimately balancing the Th1/Th2 immune response. Consequently, this effect leads to an improvement in gastric mucosa inflammation.

## 4 Discussion

*Helicobacter pylori* is a unipolar, multi-flagellated, helical, and Gram-stain-negative bacterium, which was first identified in the human gastric mucosa by Marshall and Warren (1984). *H. pylori* releases various virulence factors, such as CagA and VacA, which further exacerbate the inflammatory response and cause sustained damage to the gastric mucosa (Kao et al., 2016). *H. pylori* can also form biofilms, which help reduce its susceptibility to antibiotics and develop resistance (Fauzia et al., 2020). The CagA protein, a major virulence factor produced by highly virulent strains of *H. pylori*, not only causes a strong inflammatory response but also induces cellular alterations. It also reduces cell viability, causes cells to undergo proliferation or apoptosis, and alters tumor suppressor mechanisms in the gastric epithelium (Sharndama and Mba, 2022). This is a significant factor leading to atrophic gastritis and gastric cancer. Another major virulence factor is VacA, which can disrupt intracellular lysosomal vesicular transport and impair the autophagy pathway (Abdullah et al., 2019), causing vacuolization of host cells, which leads to cell necrosis. It can also induce apoptosis in gastric epithelial cells via the mitochondrial pathway.

The *in-vitro* experiments showed that the eradication rate of the quadruple therapy was slightly higher than that of the three compounds (JF-1, 2, and 3) and the mixture (JF-4). However, the highest eradication rate was observed with the high-dose 6-gingerol standardized product (SS). The best improvement, as evidenced by serum and gastric pathology tests, was observed with the positive drug, while the effect of high-dose 6-gingerol and its derivatives was comparable to that of the positive drug. 6-gingerol and its derivatives can effectively inhibit the growth of *H. pylori in vitro*. It is hypothesized that the eradication of *H. pylori* may be dependent on high-dose 6-gingerol and its derivatives. However, the safety of these compounds needs to be verified in both animals and human studies. 6-Gingerol and its derivatives are effective in inhibiting the growth of *H. pylori in vitro*.

*Helicobacter pylori* infection induces a certain degree of immune response in the host, releasing numerous inflammatory mediators, exacerbating gastric mucosal damage, and leading to gastroduodenal-related diseases. There is abundant evidence (Laky et al., 2015; Bagheri et al., 2016; Xie et al., 2020) that CD4+ T helper cells are activated in the immune response against *H. pylori* infection, differentiating into various effector cells (including Th1, Th2, Th17, and Treg) and producing characteristic cytokines. Among them, Th1 cells characteristically secrete IFN-γ, IL-2, and TNF-α, which mediate cellular immune responses, while Th2 cells secrete IL-4, IL-11, and IL-13, which mediate humoral immunity (Yang et al., 2021). There are reciprocal inhibition and transformation among them.

**Figure 7:**
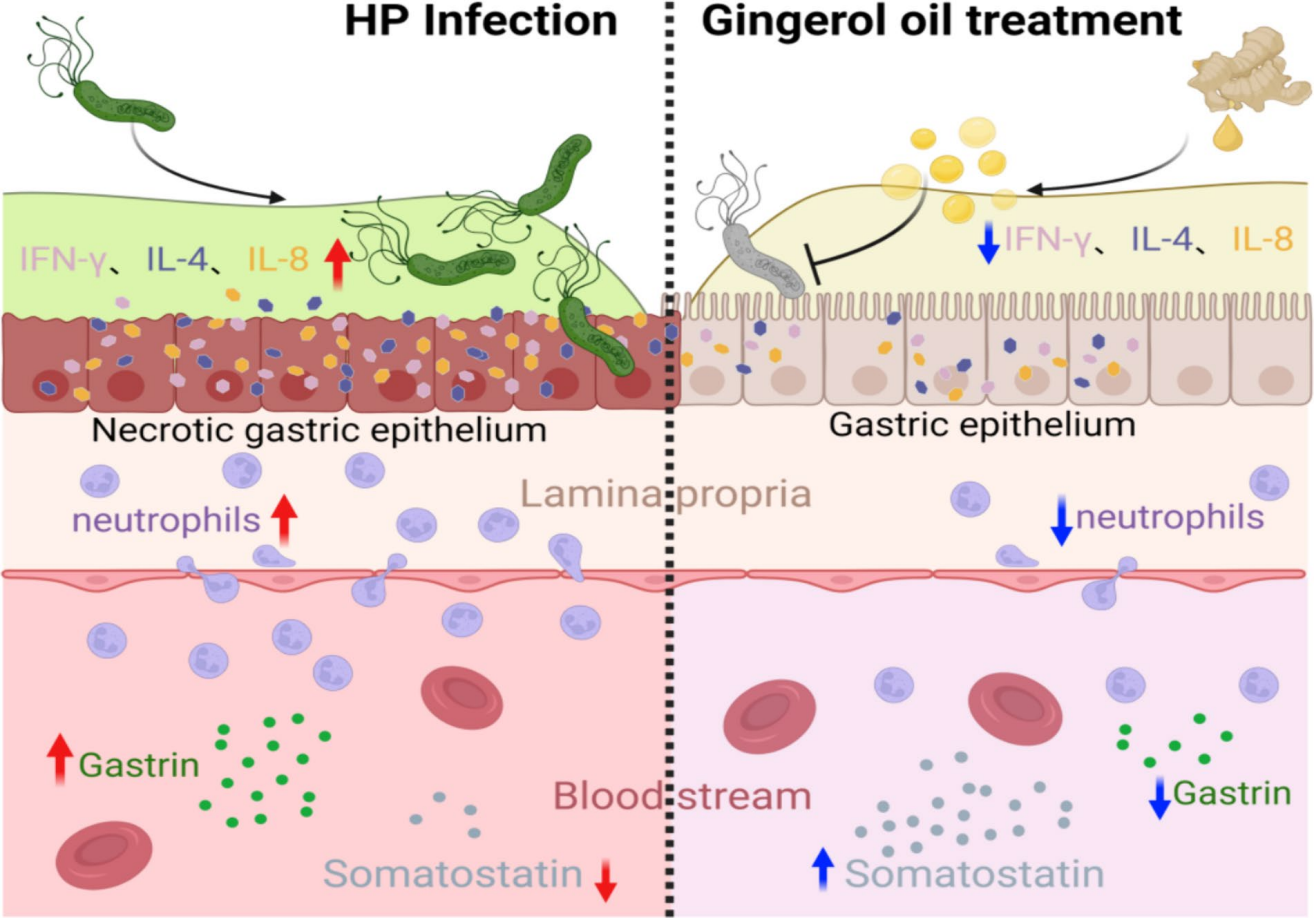
6-Gingerol treatment alleviates gastric epithelial inflammation after *H. pylori* infection, promotes the secretion of somatostatin, and inhibits gastrin. (Image produced by https://www.biorender.com/).

The expression of IL-8 increases in the host after the *H. pylori* infection (Zhang et al., 2019; Yu et al., 2020). Several studies (Aktan et al., 2006; Saha et al., 2016) have shown that ginger extracts, including 6-gingerol, inhibit the activation of NF-κB induced by many drugs and downregulate gene products involved in the NF-κB regulation of cell proliferation and angiogenesis, including IL-8. It is suggested that *H. pylori* infection may trigger the secretion of IL-8 from gastric epithelial cells by activating the NF-κB pathway, leading to the recruitment of inflammatory cells, and these compounds may block this pathway (Figure 7).

## 5 Conclusion

In summary, 6-gingerol and its derivatives could effectively inhibit the growth of *H. pylori in vitro*. In light of the high MIC values obtained, it is speculated that the eradication of *H. pylori* may depend on high doses of 6-gingerol and its derivatives. The safety of these compounds must be confirmed in animal and human studies. When used alone, 6-ginger hydroxybenzene or its derivatives have a low eradication rate of *H. pylori*, but they can still effectively suppress *H. pylori* proliferation, improve gastric mucosa inflammation, reduce inflammatory cytokines, balance the Th1/Th2 immune response, and restore gastric secretion elements and somatostatin levels. Therefore, 6-gingerol and its derivatives as a new auxiliary for *H. pylori* infection gastritis treatment strategies help to improve gastritis and reduce the occurrence of tumors. In addition, the combination of antibiotics and PPI can improve the eradication rate of *H. pylori* and reduce side effects.

This study has certain limitations. First, our study was only confined to the macro-level analysis of drug inhibition of *H. pylori*. The specific inhibition mechanism needs to be further studied, and the identification method of *H. pylori* infection needs to be further improved. Second, among the many phenotypes in gastric inflammation, the study only detected Th1/Th2-related characteristic indicators, rendering the analysis incomplete to a certain extent. Based on the findings of this study, it is suggested that future studies should explore the following aspects: the specific mechanism of 6-gingerol and its derivatives to inhibit *H. pylori* and whether these compounds can improve other stomach diseases, such as gastric cancer, *in vivo*. Exploring these research avenues not only complement the shortcomings of existing research but also lead to more valuable discoveries.

## Data availability statement

The datasets presented in this study can be found in online repositories. The names of the repository/repositories and accession number(s) can be found in the article/supplementary material.

## Ethics statement

The animal study was approved by the Ethics Committee of the Animal Experimentation Center of Yantai University. The study was conducted in accordance with the local legislation and institutional requirements.

## Author contributions

JQ: Conceptualization, Data curation, Investigation, Validation, Writing – original draft, Writing – review & editing. ZL: Data curation, Formal analysis, Project administration, Software, Writing – original draft, Writing – review & editing. JW: Data curation, Formal analysis, Writing – review & editing. YL: Conceptualization, Formal analysis, Investigation, Project administration, Supervision, Writing – original draft, Writing – review & editing. YY: Formal analysis, Funding acquisition, Methodology, Project administration, Resources, Supervision, Writing – original draft, Writing – review & editing.
